# Diarrhea-associated Hemolytic Uremic Syndrome in Adults: Two Case Reports and Review of the Literature

**DOI:** 10.7759/cureus.4435

**Published:** 2019-04-11

**Authors:** Yadav Pandey, Dinesh Atwal, Appalanaidu Sasapu

**Affiliations:** 1 Department of Internal Medicine, University of Arkansas for Medical Sciences, Little Rock, USA; 2 Department of Hematology and Oncology, University of Arkansas for Medical Sciences, Little Rock, USA

**Keywords:** hemolytic uremic syndrome, hemolytic anemia, thrombotic microangiopathy syndrome

## Abstract

Hemolytic uremic syndrome (HUS) is a type of thrombotic microangiopathy syndrome (TMA) defined as a triad of non-immune microangiopathic hemolytic anemia, thrombocytopenia, and acute kidney injury. Shiga toxin (Stx) or diarrhea-associated HUS is one of the major categories of secondary HUS, which is seen predominantly in children and is regarded as a rare entity in the adult population. We present two cases of sporadic Stx or diarrhea-associated HUS in adult females. Our first case is a 74-year-old Caucasian woman who presented to the emergency department with nausea, vomiting, and bloody diarrhea for five days. The patient reported a history of consuming meatloaf from a local store three days prior to the onset of symptoms. On presentation, laboratory workup was consistent with hemolytic anemia, thrombocytopenia, and acute kidney injury. Thrombocytopenic purpura was ruled out with normal ADAMTS13 activity. The patient’s kidney function improved and the platelet count recovered to normal with supportive measures and did not require renal replacement therapy. In the second case, we describe a 79-year-old Caucasian woman with a history of metastatic lung cancer who presented with abdominal pain, nausea, vomiting, and bloody diarrhea. History was positive for consuming meat from a local restaurant a day prior to the onset of symptoms. Initial laboratory work showed severe thrombocytopenia, microangiopathic hemolytic process, and acute kidney injury requiring continuous renal replacement therapy. Due to the unfavorable prognosis of her metastatic lung cancer, the patient and the family members decided to opt for hospice care and she was subsequently transferred to the inpatient hospice.

Diarrhea-associated HUS or Stx-HUS is a relatively underreported entity among the adult population. The treatment of typical or Stx-HUS is mainly supportive, but it is critical to rule out other causes of TMAs, especially thrombotic thrombocytopenic purpura (TTP), as it is a medical emergency that requires prompt plasmapheresis.

## Introduction

Hemolytic uremic syndrome (HUS) is a type of thrombotic microangiopathy syndrome (TMA), which is described as a combination of non-immune microangiopathic hemolytic anemia, thrombocytopenia, and acute kidney injury [1]. HUS is further divided into primary (or atypical) and secondary HUS. Shiga toxin (Stx) or diarrhea-associated HUS is one of the major categories of secondary HUS, which is a well-known entity seen predominantly in children [2]. Our experience with Stx-HUS in adults was primarily anecdotal until the largest known outbreak of Shiga-like toxin producing Escherichia coli (STEC) in Germany (2011). The outbreak unusually affected a large number of a predominantly female adult population [3]. Outside of this outbreak, we still have limited experience with Stx-HUS in adults. We present two cases of sporadic Stx or diarrhea-associated HUS in adult females.

## Case presentation

Case 1

A 74-year-old Caucasian woman presented to the emergency department with nausea, vomiting, and bloody diarrhea for five days. She had presented to her primary care physician on the third day of illness and was treated with supportive measures for presumed viral gastroenteritis. Her nausea and diarrhea improved, but she continued to have poor oral intake and increased weakness. The patient reported a history of consuming meatloaf from a local store, three days prior to the onset of symptoms. On presentation to the emergency department, vital signs were stable and physical examination was remarkable only for dry mucous membranes. Initial laboratory findings showed hemoglobin of 12 g/dl (which was reduced from 15.4 two months ago), white blood cell (WBC) 8.8 x 103/mL, platelet of 47 x 103/mL (which was lower than 161 two months ago), sodium of 119 mmol/L, potassium 4.4 mmol/L, creatinine of 6.7 mg/dl (baseline creatinine was 1.0 mg/dl), and blood urea nitrogen of 99 mg/dL. Urinalysis was concerning for urinary tract infection. A peripheral blood smear showed mildly increased schistocytes (2/high power field), normochromic normocytic erythrocytes, and marked thrombocytopenia. Hemolytic workup was significant for an elevated reticulocyte count of 3.08%, lactic acid dehydrogenase (LDH) elevated to 480 IU/L (normal: 110-240 IU/L), normal bilirubin, haptoglobin of 163 mg/dL (normal: 30-200 mg/dL), and free hemoglobin was elevated to 115 mg/dL (normal: <10 mg/dL). Urine culture grew Enterococcus faecalis, and the patient received ampicillin.

Due to concerns of new-onset thrombocytopenia, anemia, and acute kidney injury, TMA was suspected. Further evaluation of TMA showed normal ADAMTS13 activity (reported as 92%), C3 complement, C4 complement, and complement CH50 (Table 1). The atypical hemolytic uremic syndrome panel was negative for any known mutations. Stool sample for culture and Shiga-like toxin could not be obtained, as the patient was constipated during the hospital stay. Nephrology was consulted for the possible need for renal replacement therapy. Despite significantly worsened renal function, the patient did not develop anuria or oliguria. The patient’s kidney function improved with supportive measures and did not require renal replacement therapy measures. Platelet count recovered to 186 x 103/mL on the fourth day of admission and her creatinine continued to improve after she was discharged. At two-months follow-up in the clinic, the patient was asymptomatic with creatinine 1.33 mg/dl and normal hemoglobin and platelet counts.

**Table 1:** Significant laboratory values on presentation and evolution of laboratory parameters over time BUN: blood urea nitrogen; LDH: lactic acid dehydrogenase; PT: prothrombin time; INR: international normalized ratio; aPTT: activated partial thromboplastin time

Day of admission	Day 1	Day 2	Day 3	Day 4	Day 5	Day 6	Day 7
Hemoglobin (g/dL)	12.0	12.5	12.0	11.4	11.6	12.0	13.1
Platelet (10^3^/mL)	47	53	128	186	226	272	285
Creatinine (mg/dL)	6.7	6.9	7.1	7.2	6.5	5.7	5.3
BUN (mg/dL)	99	97	100	98	92	72	63
LDH (IU/L)	480						
Haptoglobin (ref: 30-200 mg/dL)	163						
Free hemoglobin (ref: <10 mg/dL)	115						
Reticulocyte count (ref: 0.5-2.1 %)	3.08						
Peripheral blood smear	Marked thrombocytopenia with increased schistocytes (2%)						
PT (ref: 10.2 -12.9 sec)	10						
INR	0.9						
aPTT (ref: 25.1 – 36.5 sec)	22.6						
Fibrinogen (ref:200 – 393 mg/dL)	454						
ADAMTS13 activity (ref: >70%)	92						
C_3 _complement (ref: 90-180 mg/dL)	105						
C_4 _Complement (ref: 15-45 mg/dL)	18.7						

Case 2

A 79-year-old Caucasian woman was transferred from an outside hospital facility with complaints of diffuse crampy abdominal pain, nausea, vomiting for three days, and diarrhea associated with blood for a day. The patient also complained of shortness of breath, decreased urine output, and swelling of lower extremities. She denied a history of fever, mental status change, or recent sick contacts. She reported a history of consuming meat from a local restaurant a day prior to the onset of symptoms. Past medical history was significant for metastatic non-small cell lung cancer and she was on treatment with osimertinib for months. On presentation, the patient was afebrile, with a blood pressure of 105/64 mmHg, heart rate of 108 bpm, respiratory rate of 16 per minute, and saturation of 98% on room air. The physical examination was significant for dry mucous membranes, distended abdomen with generalized tenderness, and pitting edema on bilateral lower extremity extending up to the knees. Initial laboratory work showed white blood cell count of 7.5 x 103/mL, hemoglobin of 11 g/dL, and platelet of 19 x 103/mL. The basal metabolic panel showed sodium of 129 mmol/L, potassium 4.1, chloride 96, bicarbonate 18, anion gap 15, blood urea nitrogen (BUN) 42 mg/dl, and creatinine 2.01 mg/dl. The peripheral blood smear revealed normocytic normochromic anemia with two to four schistocytes/hpf and severe thrombocytopenia supporting the microangiopathic hemolytic process. Subsequent workup showed elevated indirect bilirubin (total 2.7 mg/dL and direct bilirubin 0.7 mg/dL), elevated lactate dehydrogenase of 994 IU/L, and negative direct Coombs test (Table 2). Abdominal imaging did not show any evidence of an acute abdominal process.

**Table 2:** Significant laboratory findings at admission and evolution of laboratory parameters over time BUN: blood urea nitrogen; LDH: lactic acid dehydrogenase; PT: prothrombin time; INR: international normalized ratio; aPTT: activated partial thromboplastin time

Day of admission	Day 1	Day 2	Day 3	Day 4	Day 5	Day 6
Hemoglobin (g/dL)	11.0	9.2	9.8	10.1	9.7	8.8
Platelet (k/mL)	19	27	25	17	12	40
Creatinine (mg/dL)	2.01	3.40	4.2	5.2	3.8	2.8
BUN (mg/dL)	42	51	65	56	40	46
LDH (IU/L)	994					
Haptoglobin (ref: 30-200 mg/dL)	<4					
Bilirubin (mg/dL)	2.7					
Reticulocyte count (ref: 0.5-2.1 %)	2.95					
Peripheral blood smear	Normocytic normochromic anemia, 2-4% schistocytes and marked thrombocytopenia					
PT (ref: 10.2 -12.9 sec)			13.4			
INR			1.2			
aPTT (ref: 25.1 – 36.5 sec)			28.6			
Fibrinogen (ref:200 – 393mg/dL)			322			
ADAMTS13 activity (ref: >70%)			92			

The patient was admitted to the medical intensive care unit for concerns of TMA and suspected sepsis. She was empirically started on broad-spectrum antibiotics. The following day, the patient became hypotensive, requiring vasopressor support, and her renal function deteriorated with anuria. Nephrology initiated continuous renal replacement therapy and the vasopressor medications were weaned off over the next two days. Her ADAMTS13 activity resulted in 92% and an atypical HUS panel could not be sent. The stool culture and stool test for enterohemorrhagic E. coli (EHEC O157:H7) performed outside the hospital were negative. Due to the unfavorable prognosis of her metastatic lung cancer, the patient and the family members decided to opt for hospice care, and she was subsequently transferred to the inpatient hospice. The patient later passed away at the hospice facility.

## Discussion

Thrombotic microangiopathy (TMA) is a diverse syndrome defined by common clinical and pathological features. It is characterized by microangiopathic hemolytic anemia (MAHA), thrombocytopenia, and end-organ damage, usually in the form of acute kidney failure [4]. Primary TMA must be distinguished from other systemic causes of the microangiopathic process, as treatment and prognosis differ among these two classes. Some of the important systemic disorders causing MAHA and thrombocytopenia are pregnancy-related complications (severe preeclampsia, eclampsia, and HELLP (hemolysis, elevated liver enzyme levels, and low platelet levels) syndrome), systemic infections, malignancies, autoimmune diseases (such as systemic lupus erythematosus, systemic sclerosis, and antiphospholipid syndrome), hematopoietic stem cell transplant, or solid organ transplant [4]. Although different nomenclatures are used by clinicians for the spectrum of TMA syndromes, it can be broadly classified into thrombotic thrombocytopenic purpura (TTP) and hemolytic uremic syndrome (HUS) [5]. TTP is caused by the severely reduced activity of von Willebrand factor-cleaving protease ADAMTS13. It is a medical emergency requiring prompt plasmapheresis and carries a very poor prognosis if treatment is delayed [6]. HUS can be further subdivided into primary (atypical) and secondary (typical) HUS. Typical HUS, also known as diarrhea-associated HUS, is commonly associated with Shiga toxin-like producing Escherichia coli (STEC) whereas atypical HUS is secondary to complement dysregulation and activation [2-5]. It is important to distinguish atypical HUS from typical HUS, as these patients might benefit from plasmapheresis or plasma infusion early during the course of illness and we have a specific treatment available for atypical HUS in the form of anti-complement therapy (eculizumab) [7].

Diarrhea-associated HUS or Stx-HUS occurs after infection with Shiga toxin-producing Escherichia coli (STEC) or less frequently after a Shigella dysenteriae infection. Enterohemorrhagic E. coli (EHEC O157:H7) is the most common cause of Stx-HUS in the United States (US). It is common in children of four years or less and relatively rare in the adult population. Infection of EHEC occurs following the consumption of contaminated, undercooked meat, unpasteurized milk, water, fruits, and vegetables [8]. The largest outbreak of EHEC infection (EHEC 0104:H4) associated HUS occurred in northern Germany in 2011, which involved 845 confirmed cases of HUS unusually affecting a large number of the adult population (88%) with a female predominance (68%) [3,9]. Excepting this outbreak, our experience with Stx-HUS in adults is still anecdotal at large. A French study by Joseph et al. has described a total of 236 patients with TMAs in a retrospective review over a course of 14 years and only 12 patients (5%) had Stx-HUS, which clearly demonstrates its rarity in the adult population [10]. In HUS, prodromal illness with nausea, vomiting, abdominal pain, and bloody diarrhea precedes the classic triad of microangiopathic hemolytic anemia (MAHA), thrombocytopenia, and acute kidney injury [11]. MAHA is defined as non-immune (Coombs test negative) hemolysis with the presence of schistocytes on peripheral blood smear and laboratory findings of hemolysis, viz., elevated LDH, indirect bilirubin, plasma-free hemoglobin, and low haptoglobin [12]. Thrombocytopenia is characterized by a platelet count of less than 140,000/mm^3^. Renal involvement in HUS ranges from mild proteinuria and hematuria to severe acute kidney injury, requiring renal replacement therapy [13].

The diagnosis of Stx-HUS is generally made on clinical and laboratory findings. Tests for the evidence of STEC infection include stool culture and testing for Shiga toxin in stool samples [14]. Stool cultures can be unreliable, as bacteria are present in stool for a few days and even direct stool testing for Shiga toxins can be insensitive in areas with low prevalence [15-16]. Both of our patients had the classic triad of HUS preceded by prodromal symptoms of nausea, abdominal pain, and bloody diarrhea that started after the consumption of meat from a local store. We could not establish the presence of STEC in our patients, as the first patient could not provide a stool sample and the second patient’s stool collected on the fifth day of illness tested negative for STEC by stool culture and direct stool antigen testing. Failure to establish STEC infection in diarrhea-associated HUS is relatively common, as reported by Mody et al., in which they could not detect STEC infection in 235 (30%) out of 770 children with Stx or diarrhea-associated HUS [17].

The treatment of typical or Stx-HUS is mainly supportive, but it is critical to rule out other causes of TMAs, especially TTP and atypical HUS, based on clinical presentation and laboratory findings [4]. Our patients presented with a typical prodromal illness preceded by the consumption of possibly contaminated meat. Despite a very low likelihood, we did rule out TTP in our patients. Complement studies and a gene mutation panel were also sent for the first patient, which were negative, but the second patient elected to go for hospice before we could send these studies. Since typical HUS is much rarer than TTP and atypical HUS in the adult population [10], ruling out these etiologies is of paramount significance. An important part of the supportive approach in Stx-HUS is renal replacement therapy, which may be needed temporarily in greater than 70% patients, as evidenced in the outbreak in Germany [3]. The use of antibiotics is controversial and the role of anti-complement therapy is uncertain [13]. With supportive management, most patients recover well, as evidenced in our first patient. Questions regarding long-term sequelae from Stx-HUS in adults are still unanswered due to the paucity of data, and we eagerly await results from the long-term follow-up of patients in the STEC outbreak in Germany.

## Conclusions

Diarrhea-associated HUS or Stx-HUS is a relatively underreported entity among the adult population. Prompt recognition and the ruling out of other TMAs is absolutely imperative.
